# First steps: study protocol for a randomized controlled trial of the effectiveness of the Group Family Nurse Partnership (gFNP) program compared to routine care in improving outcomes for high-risk mothers and their children and preventing abuse

**DOI:** 10.1186/1745-6215-14-285

**Published:** 2013-09-08

**Authors:** Jacqueline Barnes, Dipti Aistrop, Elizabeth Allen, Jane Barlow, Diana Elbourne, Geraldine Macdonald, Edward Melhuish, Stavros Petrou, Joshua Pink, Claire Snowdon, Helen Spiby, Jane Stuart, Joanna Sturgess

**Affiliations:** 1 Birkbeck, University of London, London, UK; 2 Sheffield Children’s NHS Foundation Trust, Sheffield, UK; 3 London School of Hygiene and Tropical Medicine, London, UK; 4 Warwick Medical School, University of Warwick, Warwick, UK; 5 Queen’s University Belfast, Belfast, UK; 6 University of Nottingham, Nottingham, UK; 7 Institute for the Study of Children, Families and Social Issues, Department of Psychological Sciences, Birkbeck, University of London, Malet Street, London WC1E 7HX, UK

**Keywords:** Early intervention, Child abuse, Nurse, Young parents

## Abstract

**Background:**

Evidence from the USA suggests that the home-based Family Nurse Partnership program (FNP), extending from early pregnancy until infants are 24 months, can reduce the risk of child abuse and neglect throughout childhood. FNP is now widely available in the UK. A new variant, Group Family Nurse Partnership (gFNP) offers similar content but in a group context and for a shorter time, until infants are 12 months old. Each group comprises 8 to 12 women with similar expected delivery dates and their partners. Its implementation has been established but there is no evidence of its effectiveness.

**Methods/Design:**

The study comprises a multi-site randomized controlled trial designed to identify the benefits of gFNP compared to standard care. Participants (not eligible for FNP) must be either aged < 20 years at their last menstrual period (LMP) with one or more previous live births, or aged 20 to 24 at LMP with low educational qualifications and no previous live births. ‘Low educational qualifications’ is defined as not having both Maths and English Language GCSE at grade C or higher or, if they have both, no more than four in total at grade C or higher. Exclusions are: under 20 years and previously received home-based FNP and, in either age group, severe psychotic mental illness or not able to communicate in English. Consenting women are randomly allocated (minimized by site and maternal age group) when between 10 and 16 weeks pregnant to either to the 44 session gFNP program or to standard care after the collection of baseline information. Researchers are blind to group assignment.

The primary outcomes at 12 months are child abuse potential based on the revised Adult-Adolescent Parenting Inventory and parent/infant interaction coded using the CARE Index based on a video-taped interaction. Secondary outcomes are maternal depression, parenting stress, health related quality of life, social support, and use of services.

**Discussion:**

This is the first study of the effectiveness of gFNP in the UK. Results should inform decision-making about its delivery alongside universal services, potentially enabling a wider range of families to benefit from the FNP curriculum and approach to supporting parenting.

**Trial registration:**

ISRCTN78814904.

## Background

Recent estimates show that suboptimal parenting of infants is a major public health issue. As of 31 March 2012, infants (children aged up to one year) accounted for 13% of those who were subject to a child protection plan in England [1]. The most common initial category of abuse for infants was neglect (49%) followed by emotional abuse (22%) and physical abuse (16%). Infants also face four times the average risk of homicide, perpetrators being parents in most cases [2]. Non-accidental head injuries are high resulting in up to 30% mortality and significant neurological impairment for survivors [3]. Furthermore, abuse of very young children may be up to 25% higher than indicated by official estimates [4]. In addition to preventing childhood injury and abuse, sensitive caregiving during the first year is important for promoting optimal child outcomes because brain development then is rapid and vulnerable to negative influences [5]. Trauma and adverse parent–child interactions in infancy elevate cortisol, a strong indicator of stress, and can lead to attachment difficulties, hyperactivity, anxiety and impulsive behavior [6,7].

Overall, evidence concerning the effectiveness of home visiting programs in reducing child maltreatment is inconclusive [8] but the Nurse Family Partnership (NFP) was one of nine home visiting programs identified by the United States Department of Health and Human Services (HHS) as part of their Home Visiting Evidence of Effectiveness (HomVEE) review. The NFP was found to be effective in both decreasing child maltreatment and improving parenting practices [9]. Long-term follow-up of the FNP suggests a 48% reduction in cases of child abuse and neglect by age 15 [10].

The NFP curriculum has strong theoretical underpinnings, both in terms of risk and protective factors, and the mechanisms through which change may be produced [11], drawing on ecological [12], self-efficacy [13] and attachment [14] theories. Ecological theory emphasizes the importance of interactions between the characteristics of individuals and their contexts; self-efficacy theory focuses on an individual’s beliefs that they can successfully carry out behavior required for good outcomes; and attachment theory highlights the importance of the early interactions with the primary caregiver in terms of the child’s later capacity for affect regulation. The cornerstone of the NFP model is the therapeutic nurse-client relationship. Beneficial outcomes found in the US trials include improved prenatal health, fewer childhood injuries, fewer subsequent pregnancies, increased intervals between births, increased maternal employment and improved school readiness [10,15-18]; it has also been shown to have the potential to be cost effective [19]. Results from the US trials of NFP found that it was particularly beneficial for women with ‘low psychological resources’, namely a combination of lower intelligence, mental health problems and low self-efficacy [20]. NFP is now being offered in England, where it is known as the Family Nurse Partnership (FNP), and has been successfully implemented across a range of settings [21-23]. It is currently available in more than 50 locations with further expansion planned [24]. There are, however, mothers-to-be who might benefit from the FNP program but who are not eligible because they are teenage and already had a previous live birth or are over the age of 19 [25]. Group FNP (gFNP) was designed as a program that could be offered to woman not eligible for FNP.

### Group Family Nurse Partnership

Group FNP was developed in 2009, jointly by the UK FNP National Unit and the US NFP National Office led by Professor David Olds, as a way to use the expertise of the FNP nurses and the learning from the FNP to reach women whose children were at risk of poor outcomes but offered in a different context and to those not eligible for FNP. The newly developed program has the same theoretical basis as the home-based program but is delivered in a local children’s center (or similar community location). The aims of the gFNP program are to improve birth outcomes, develop a warm and authoritative parenting style underpinned by good attachment and knowledge of babies’ developmental needs, promote effective local support networks through contact with other parents, increase take-up of local services, and greater parental self-efficacy to make positive life choices and plan for their future through, for example, thinking about gaining educational qualifications or training for employment. While FNP continues until infants are 24 months old gFNP runs until infants are 12 months of age [26].

The sites delivering gFNP are established FNP sites and gFNP will be delivered in addition to the one to one FNP program in those sites, to clients who would are ineligible for FNP. The program is delivered to each group by two family nurses trained according to US NFP guidelines and who are experienced in delivering home-based FNP [26], one of whom is a practising midwife. Each group comprises between 8 and 12 women, whose partners are encouraged to attend. Women using group based antenatal care such as the Centering Pregnancy Model [27], reported a preference for this approach over traditional care [28-30]. Centering Pregnancy has been associated with improved prenatal outcomes such as reductions in preterm births among high-risk women [31,32]. Following this model of delivery, gFNP provides routine midwifery care in pregnancy and infant health checks according to NICE guidelines [33] during the gFNP session with encouragement for mothers to conduct the necessary pregnancy checks themselves with guidance from the family nurses and midwives. Any out-of-hours antenatal care is managed according to local arrangements (this may involve a community midwifery team or hospital) and intrapartum care is managed by the hospital/birth center midwifery team. The group context should help mothers to develop social networks with other local mothers [34], and to benefit from peer-group learning. Also, meeting in Children’s Centers helps families to become familiar with the other services and to develop regular contact with other professionals when additional support is called for [35].

An implementation evaluation in England found that both the mode of delivery and materials were acceptable to clients and nurses [34-36]. In addition to developing relationships with the family nurses, clients have developed close relationships with group members and made regular use of other services in the Children’s Centers. The delivered content of gFNP is consistent with NFP US National Office recommendations [36] and the prediction is that gFNP is likely to be associated with the same kinds of benefits as the individually delivered program. However, in order to provide optimal information for service providers and commissioners, and to provide the most effective support for potentially vulnerable families, it is important that programs be subject to rigorous randomized trials to provide evidence about efficacy and cost-effectiveness.

### Aims of the study

The main objective of this study is to evaluate whether provision of the Group Family Nurse Partnership (gFNP) program, compared to routine antenatal and postnatal services, can enhance parenting and reduce risk factors for maltreatment in expectant mothers aged < 20 with one or more previous live births or expectant mothers aged 20 to 24 with low/no educational qualifications and no previous live births.

Secondary objectives are to determine whether provision of gFNP will enhance maternal physical and mental health in pregnancy and the experience of pregnancy and delivery for mothers and fathers; to determine whether gFNP will enhance infant birth status and health status in infancy, breast feeding in the first two months, and immunization take-up during the first year; to identify whether gFNP is cost effective in comparison with routinely available services; and to identify how the program is experienced by women who have a history of being in the care of social services (known as having been ‘looked after’) in comparison with receiving routinely available services.

## Methods/Design

### Study design

The study comprises a multi-site randomized controlled trial in which eligible women are allocated (minimized by site and maternal age group) to one of two arms: i) gFNP delivered via 44 sessions delivered over 76 weeks (N = 100); ii) standard care (N = 100). In addition to the quantitative measures used to compare the two groups qualitative methods will be used to provide a better understanding of the results obtained in the trial.

### Study setting

Sites were included if they responded to an invitation to take part from the FNP National Unit, could demonstrate sufficient women of the relevant age and parity who had given birth in the previous year, and could confirm good links with community midwifery, who also had to sign the expression of interest. Only FNP teams who had delivered the home-based FNP program in its entirety (from birth to child age 24 months) to a cohort of women and with a practising midwife in the team were eligible.

The gFNP program is delivered by two family nurses, one of whom is a practising midwife, in community settings such as Sure Start Children’s Centers, located so that the amount of travelling necessary by group participants is kept to a minimum. Proximity to the location should enhance the rate of attendance and also foster the formulation of social networks. However, so that women from a potentially wider range of home addresses can be recruited to ensure the sample size, funds are available to reimburse intervention participants for travel by public transport to the intervention sessions. Child care is available for any other children. The gFNP program runs from approximately the 16th week of pregnancy to when the babies are 12 months old, meeting weekly or fortnightly during the daytime depending on the stage of the program (14 pregnancy sessions and 30 infancy sessions).

The control group mothers and infants would not be eligible for home-based FNP but will receive all the Universal Core Offer recommended in the Healthy Child Program through midwifery and health visiting services [37]. This is a substantial and universal service for expectant mothers and their infants with a strong evidence base [38] supported by systematic review evidence and NICE guidance. It offers screening tests, immunizations, developmental reviews, information and guidance to support parenting. The latest version has a strong emphasis on pregnancy and the first year of life. Community midwives and health visitors may provide or refer to a range of other community-based or specialist services designed to support young mothers.

Data collection for the study will be conducted by researchers blind to allocation in three visits to participant’s homes (baseline in pregnancy, when infants are two months and twelve months of age), and one telephone contact (when infants are six months old).

### Ethical and research governance approval

The study has been granted ethical approval by the NRES Committee South West-Frenchay (REC reference 13/SW/0086).

### Study timeline

**Figure 1:**
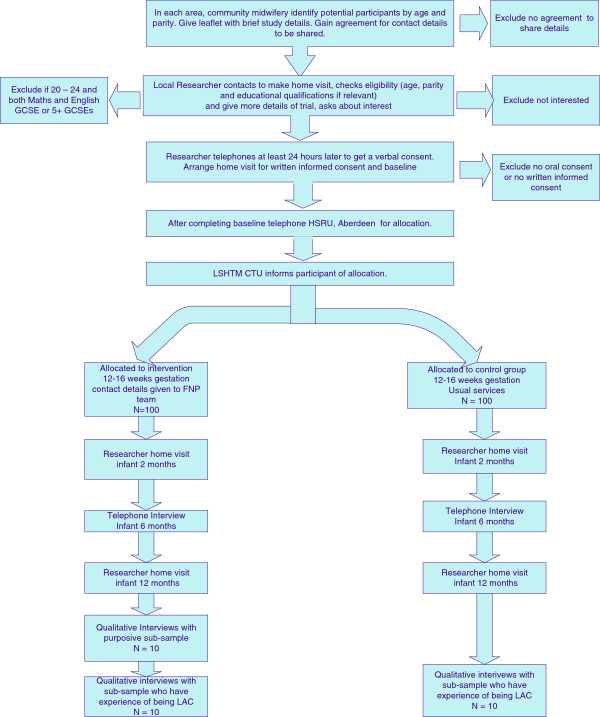
Flow chart of participants.

The trial commenced in February 2013 and will be completed in January 2016. Recruitment commenced in July 2013 and takes place in seven sites in England (Barnsley, Dewsbury, Lewisham, Nottingham, Sandwell, South Tyne and Wear and Waltham Forest) through until January 2014. Figure 1 provides details of the timeline for each participant, which will extend from the time that they book in with their midwife (on average at 8 to 12 weeks gestation) through to the end of the study, when their infants are at least 12 months old. The specific time will vary depending on their gestation at booking but will range from 82 to 86 weeks. The intervention arm will receive the gFNP program for 76 weeks on average (from 16 weeks gestation to infant 12 months).

### Randomization

The process will be overseen by the London School of Hygiene and Tropical Medicine Clinical Trials Unit (LSHTM CTU). Identifying information and baseline details of eligible consenting mothers-to-be will be provided by the researchers to the central randomization service at Health Service Research Unit (HSRU), Aberdeen. Minimization criteria will be used to ensure a balance of key prognostic factors using the following two criteria: site and age group (< 20, 20 to 24 years). Allocations to one of two arms will be securely computer generated and delivered by Email to LSHTM. The LSHTM CTU will convey to each gFNP team the names and contact details of women allocated to the intervention arm. The researchers will be blind to allocation.

### Study participants

Women eligible for the trial are expectant mothers prior to 16 weeks gestation with expected delivery dates (EDD) within six to eight weeks of each other for each group in each site, with the range of EDDs specified in relation to the expected date of the first meeting for that site so that the majority would have a gestation of 16 to 20 weeks when the program commences. Specific criteria beyond similar EDDs and gestation were developed based on a formative evaluation [34-36] and on the requirement of the UK FNP National Unit that gFNP should be offered to women not eligible for FNP but be likely to benefit from the content of program, based on research in the USA [11,15]. Thus participants must be either aged < 20 at their last menstrual period (LMP) with one or more previous live births, or aged 20 to 24 at LMP with low educational qualifications and no previous live births. ‘Low educational qualifications’ is defined as not having both Maths and English Language GCSE at grade C or higher or, if they have both of these GCSEs, no more than four GCSEs in total at grade C or higher. Exclusions are: expectant mothers under the age of 20 who have previously received home-based FNP, those in either age group with psychotic mental illness (defined as bi-polar disorder or schizophrenia), or those who are not able to communicate in English since it is not possible to deliver the program in a group context unless all the participants can all understand and speak English.

### Sample size

The sample size was calculated for the two primary outcomes, the revised Adult Adolescent Parenting Inventory (AAPI-2) [39] and the observational CARE Index [40-42]. The standard deviation (SD) of the AAPI-2 is 10, with differences of 6.7 identified in the normative sample between abusive and non-abusive adult females [39]. The standard deviation for the CARE Index is expected to be around 2.3 [41].

For this individually randomized trial, we propose to recruit sufficient mothers and babies (families) to allow the trial to detect a difference between groups of 0.5 standard deviations, with 90% power at a significance level of 0.05 (2-tailed); this is considered to represent a moderate size of effect [43]. Basing calculation on the AAPI-2, very conservatively assuming a correlation of 0.4 between pre and post intervention scores we would need at least 71 families in each arm of the trial to detect this difference. Allowing for an expected 30% drop out rate (based on the first two applications of the program in England) we would need to recruit a minimum of 84 families per arm of the trial. We therefore propose, conservatively, to recruit a minimum of 100 families per arm (N = 200). In the intervention arm this will consist of 10 groups of (generally) 10 families (N = 100), with 2 groups provided per site. However, given that the minimum number required for a viable group is 8, 7 sites are involved in the trial so that it will be possible to recruit a minimum of 208 (if one site proves able to deliver only one group of 8) and potentially at least 224 participants (2 groups of 8 per site plus the same number of controls). It was necessary to have two more sites than the five originally proposed to cope with the possibility that in one or more areas area the minimum number of women would not be recruited to form a viable group. The proposed sample size would similarly allow us to detect a change of approximately 0.5 standard deviations in the CARE index [40-42]. We would therefore expect to be able to detect a difference at follow-up between arms of the trial of approximately 1.2 with 90% power and a 5% level of significance.

### Recruitment

Recruitment will be time-dependent in that a minimum number of trial participants with expected delivery dates (EDDs) that are within 8 weeks of each other must be recruited within 6 weeks in each site for each delivery of gFNP, namely at least 16 and ideally 20 (8 to 10 intervention and 8 to 10 control) with a maximum number per group of 12. In the recruitment process, community midwives will identify women using the initial criteria of age, parity and gestation. Guidelines will also be provided about the specific EDDs that are required for each gFNP delivery in each site.

Midwives will give all potentially eligible women a brochure describing the study, asking for agreement to give their names and contact details to the local researcher as part of a staged consent process, using an ‘agreement to contact’ form. Those agreeing will be contacted by the local researcher. The first contact will be by telephone to establish eligibility. Women who are not eligible will be thanked for their time. Those who are eligible will be given further information about the trial, and time to think about participation. After at least 24 hours, the researcher will contact the women by telephone and if telephone agreement is given the researcher will make a home visit, obtain written consent and collect baseline data. Participants will be reminded about the services available in their area and that they should keep their next midwifery appointment and remind them about the next research contact. After leaving, the local researcher will telephone the central randomization service provided by HSRU Aberdeen to randomize the participant, giving identifying details and sufficient prognostic detail to allow minimization. Researchers will be given a study number for the participant but will remain blind to allocation, which will be conveyed by HSRU Aberdeen to LSHTM CTU by secure Email.

LSHTM CTU will inform all participants by first class post of their allocation to the intervention or control arm of the study. For women in the intervention arm, the LSHTM team will inform the relevant FNP team by fax, receipt confirmed by fax, of the women in the intervention arm so that the gFNP intervention can be initiated. This process will continue until at least 16 and no more than 24 women have been recruited in each site for the first delivery of gFNP. If a second group is to be offered in the same location, a second round of recruitment will take place 12 weeks later, repeating the same process.

As a strategy to limit attrition, participants will be given ‘High Street’ vouchers for £20 at each home-visit data collection point (baseline, two months postpartum and twelve months postpartum) and a £10 voucher will be posted after the six month telephone contact as acknowledgement of their contribution, and to encourage continuation with the study. In addition, details of changes of address will be solicited by providing pre-paid addressed envelopes and response cards at baseline by sending birth congratulation cards and one year birthday cards.

### Data collection instruments

#### Primary outcome measures

Two primary outcome measures of parenting are being used because of the difficulties associated with the detection of low frequency events such as child abuse. One is a self-report measure of parenting opinions and the others an objective measure of parent-infant interaction. Both are known to be able to identify mothers at risk for abusive parenting.

The revised AAPI-2 [39] is a 40 item self-report measure able to discriminate between abusive and non-abusive parents. It includes five subscales: ‘inappropriate’ expectations of children, inability to demonstrate empathy to children’s needs, strong belief in the use of corporal punishment, reversing parent–child family roles and oppressing children’s power and independence. Responses are on a five-point Likert-type scale ranging from‘strongly agree’ to ‘strongly disagree’. Internal reliability of the subscales ranges from .83 to .93, Cronbach alphas range from .80 to .92. The scales were constructed based on factor analysis to demonstrate construct validity and the inventory has discriminant validity comparing abusive and non-abusive parents (sample 1,985).

The observational CARE Index [40,41] is based on a video recording of three minutes mother-child play, and measures three aspects of maternal behavior (sensitivity; covert and overt hostility; unresponsiveness) and four aspects of infant behavior (cooperativeness; compulsive compliance; difficultness; and passivity). These are highly correlated with attachment and differentiate between abusing, neglecting, abusing and neglecting, marginally maltreating, and adequate dyads [42]. Scores range from 0 to 14, higher scores indicating better sensitivity and/or co-operation. Scoring will be conducted blind to allocation.

In addition the incidence of child abuse during the child’s first year will be assessed by the number of case conferences; the number of children with Child Protection Plan in place; and the number of children removed from the home (all based on maternal reports at 12 months). The child’s attendance at hospital A&E departments for non-accidental injuries or ingestions of toxic substances will be confirmed using hospital episode statistics (HES) data.

#### Secondary outcome measures

Secondary outcomes will assess socio-emotional aspects of parenting and family life and service use. Maternal depression will be assessed (baseline, 2, 6 and 12 months postpartum) using the Edinburgh Postnatal Depression Scale [44], a well-validated 12 item measure of postnatal depression with high reliability (0.88) and internal consistency (0.87), 86% sensitivity and 78% specificity. This questionnaire will be scored within 24 hours of its administration so that any woman with a total score above the recommended cut-off indicating a risk of depression, or who responds affirmatively to the question asking about self-harm, can be identified and a health care professional contacted to give appropriate support.

Maternal stress will be assessed (2 and 12 months postpartum) using the Abidin Parenting Stress Index, Short Form [45], a well-validated 36 item measure of perceived stress in the parenting role with sound test-retest reliability (r = .84) and internal consistency (a = .91). High scores on the PSI have been associated with abusive parenting [46,47] with some evidence that parenting stress is higher in women with five or more risk factors for child abuse [48]. In addition, parenting sense of competence will be assessed with the Parenting Sense of Competence (PSOC) scale [49] at two and twelve months. This 17 item measure has three factors; satisfaction, efficacy and interest established by factor analysis in a normative non-clinical sample, each with acceptable internal consistency (from 0.62 to 0.72) [50]. Maternal health-related quality of life (HRQoL) will be assessed using the EuroQol EQ-5D-5 L measure [51] at baseline, two, six and twelve months postpartum. This contains a visual analogue scale asking patients to rate their current HRQoL on a scale from 0 to 100, and a five dimension health status classification system, which can then be converted to a multi-attribute utility score by applying a UK tariff [52]. Brief questions designed for the study and based on those developed for use when delivering FNP will ask about maternal smoking, alcohol and drug use and relationship violence.

The extent of social support available to the mothers will be assessed (baseline and 12 months) using the Medical Outcomes Study (MOS) Social Support Survey [53]. The 20 item scale measures four dimensions of support, established using confirmatory factor analysis: emotional support, tangible support, positive interaction, and affection, each with internal consistency of 0.91 or higher, and also provided a total support score (Cronbach alpha 0.97); stability over time is also high for each scale (ranging from 0.72 to 0.78) [53]. Use of local resources and services will be assessed using questionnaires designed specifically for this study. Brief questions designed for the study will ask about infant feeding and the take-up of recommended immunizations in the infant’s first year.

#### Process measures

Data forms completed after each group session by the FNs delivering gFNP will provide information on intervention participants’ attendance at each session, the presence of partners and the extent of their understanding and involvement in the content. The ID number used by the FNs for program delivery data will be linked with the trial number.

Qualitative semi-structured interviews will be conducted with selected professionals in each locality to ascertain the extent to which the referral pathways could be strengthened, the likelihood that the service could be successfully embedded in the area and its likely sustainability. These interviews will take place after all recruitment has been completed in each local area with professionals likely, in the future, to be involved in making referrals to the program (in total, at least five community midwives and at least five other relevant health professionals such as general practitioners). At the completion of program delivery in each area, interviews will also be conducted with at least five commissioners of children’s services and with two professionals who are providers of care for ‘looked-after’ young people.

So that the research does not influence the experience of participants in the research, a number of interviews will be conducted after completion of program delivery in each group. At least one family nurse will be interviewed per center and each FNP supervisor will be interviewed about their thoughts on delivering the program and its likely sustainability. They will also be asked about the particular relevance of the gFNP program for women or their partners who have a ‘looked-after’ history or who are currently under the care of social services.

Participants’ views about the acceptability of the program will be assessed using semi-structured face-to-face interviews with a purposive sample (informed by program delivery data) of 20 women who were allocated to receive gFNP support and 5 of their partners.

In-depth semi-structured interviews will be conducted with up to 10 women allocated to receive gFNP (excluding those specified in the previous paragraph) who are or who have a history of being looked after and, if available, their partners, and a similar number of women allocated to standard care to explore their perceptions of the program, or any impacts, and other support needs.

### Data management

Quantitative data (summarized in Table 1) will be collected from both the intervention and control arms of the study by study researchers in the participants’ homes and by telephone. The data will comprise a range of self-report questionnaires. Response cards will be used for questions that have a range of possible responses. Home visits will be made at baseline, when participants’ infants are two and twelve months old with a telephone contact when infants are six months old. At 12 months postpartum, data collection will also include independent video-recorded observations of the mother and infant. Researchers will administer questionnaires orally where required with the anticipation that some participants may have low levels of literacy. Researchers will be trained together to ensure that similar methods are used in all locations and the trial manager will accompany them on a percentage of home visits throughout the study to maintain quality.

**Table 1:** Details and timing of data collection

**Measure**	**Baseline, gestation 10 to 14 weeks**	**Infant 2 months, home visit**	**Infant 6 months, telephone interview**	**Infant 12 months, home visit**
Adult-Adolescent Parenting Inventory (AAPI-2)	X			X
CARE index				X
Demographics	X	X (update)	X (update)	X (update)
Edinburgh Postnatal Depression Scale (EPDS)	X	X	X	X
Infant feeding	X (plans)	X	X	
Infant immunizations		X		X
Maternal drug use	X	X (update)		X (update)
Maternal quality of life (EQ-5D 5L)	X	X	X	X
Maternal smoking and alcohol use	X	X (update)		X (update)
Parenting stress index short form (PSI)		X		X
Perceived parenting competence (PSOC)		X		X
Relationship violence	X			X
Social networks (MOS)	X			X
Service use		X	X	X

Data will be double entered to ensure quality and a percentage of completed forms reviewed for quality. All LSHTM data will be managed and stored in compliance with ICH GCP 1996 following trial specific standard operating procedures (SOP) as given in the trial master file, and in accordance with LSHTM Information Management and Security Policy and LSHTM Data Protection Policy. Anonymized data will be stored securely at the LSHTM and separately from any information identifying participants. Paper forms will be stored in numerical order in a secure and accessible place and will be maintained in storage for a period of three years after completion of the study. Electronic data will be stored as csv files and Stata data files (.dta) in LSHTM data centers which provide appropriate levels of environmental and physical security on servers that are managed in accordance with LSHTM Systems Management Policy. Confidential information will be registered with the LSHTM Archivist and Records Manager and data will be stored on a secure server which maintains an audit trail demonstrating system access. At the end of the study, routinely collected HES data for trial participants will be accessed for information on all hospital health service activity during the period between randomization and 12 month postpartum. HES data cover hospital in-patient, outpatients, and accident and emergency attendances.

### Data analysis

#### Outcomes

Statistical analysis will be carried out at the individual level. Every effort will be made to obtain outcome measures for participants, even if some drop out during the course of the group sessions or move out of the local area so have to stop attending gFNP.

Analyses will be carried out using two different types of dataset:

i) The intention-to-treat datasets: data will be analyzed based on participants according to the random allocation irrespective of whether the intervention was or was not entirely or partly taken up.

ii) The per-protocol datasets: as it is possible that participants may not receive the intervention, the intention-to-treat analysis might underestimate the potential efficacy of intervention. A per-protocol analysis will therefore be carried out in addition to the intention-to-treat analysis. The per-protocol datasets will include data pertaining to all outcomes, restricted to those participants who complied fully or partly with their assigned intervention.

The primary analysis will use the intention-to-treat datasets. Demographic and other characteristics at trial entry will be tabulated using the intention-to-treat datasets. No significance tests will be performed to test for differences at baseline. Descriptive statistics for continuous variables will include the mean, standard deviation, median, range and the number of observations. Categorical variables will be presented as numbers and percentages.

The data will be analyzed by multiple regression modeling, with appropriate generalized linear models (GLMs) used to examine the effect of the intervention, fitting baseline measures of outcomes as covariates, where available. A small number of secondary analyses based on explicit hypotheses, for example, subgroup (including ‘looked after’ history)/explanatory analyses (considering compliance with the interventions) will be specified in advance (in a statistical analysis plan). The secondary analyses will include an analysis in which the small groups in which the intervention is delivered will be fitted as a random effect to allow for any potential clustering by group. Sensitivity analyses will be conducted for all primary outcomes. Inverse probability weighting would be considered if missing data were larger than expected and/or there was differential attrition between the trial arms. Additionally, reasons for the differential attrition will be fully explored.

#### Economic evaluation

A prospective economic evaluation, conducted from an NHS and personal social services perspective, will be integrated into the trial. The economic assessment method will, as far as possible, adhere to the recommendations of the NICE Reference Case [54]. Primary research methods will be followed to estimate the costs of the delivering gFNP, including development and training of accredited providers, the cost of delivering the group sessions, participant monitoring activities, and any follow-up/management. Broader resource utilization will be captured through two principal sources: (i) routine health service data collection systems described above; and (ii) patient questionnaires administered at baseline, two months postpartum, and twelve months postpartum with a telephone contact at six months to minimize loss of information due to recall difficulties. Unit costs for health and social care resources will largely be derived from local and national sources and estimated in line with best practice. Primary research using established accounting methods may also be required to estimate unit costs. Costs will be standardized to current prices where possible.

Maternal health-related quality of life, measured at baseline, two months postpartum, six months postpartum and twelve months postpartum using the EuroQol EQ-5D-5L [51] will be converted into health utilities using established utility algorithms [52] for the purposes of quality-adjusted life year (QALY) estimation. The results of the economic evaluation will primarily be expressed in terms of incremental cost per QALY gained. Non-parametric bootstrap estimation will be used to derive 95% confidence intervals for mean cost differences between the trial groups and to calculate 95% confidence intervals for incremental cost effectiveness ratios [55]. A series of sensitivity analyses will explore the implications of uncertainty on the incremental cost-effectiveness ratios and will consider the broader issue of the generalisability of the results. One such analysis will adopt a societal perspective incorporating direct costs to trial participants and their partners, informal care provided by family and friends, and productivity losses. In the baseline analysis, and for each sensitivity analysis, cost-effectiveness acceptability curves will be constructed using the net benefits approach [56].

More extensive economic modeling using decision-analytic methods will extend the time horizon of the economic evaluation, drawing on best available information from the literature together with stakeholder consultations to supplement the trial data. Parameter uncertainty in the decision-analytic model will be explored using probabilistic sensitivity analysis. Longer term costs and consequences will be discounted to present values using discount rates recommended for health technology appraisal in the United Kingdom [54]. Given the plethora of outcome measures across several domains for the mother, child and broader family, a separate discrete choice experiment (DCE) will be conducted. The possibility of combining the outputs of the DCE with cost estimates and changes in relevant outcomes within a cost-benefit analysis framework will be explored.

#### Process

Characteristics of study participants and those who decline to participate or are ineligible will be identified (demographic and risk profiles) and contrasted with those of the wider population in the study areas. The representativeness of recruited families will be assessed by analyzing anonymized data for each expectant woman approached: age; parity; any risk factors collected during routine booking-in (for example, history of mental health problems); in relation to their acceptance or rejection of the agreement for researcher contact.

The uptake rate of women who agree to the intervention will involve an assessment of the ratio of women randomized to receive the intervention who then attend at least one session relative to those who either refuse after meeting with the family nurse, or who agree but never attend any sessions. The study attrition rate will be estimated in terms of the proportion of women who drop out relative to those who continue in either arm of the trial and also those who may or may not take part in research visits but cease to receive the intervention, based on information provided by the nurses delivering the program. The extent to which the program is being delivered with integrity will be assessed though analysis of data from the program’s standardized data forms documenting attendance, the content domains covered in each session and participants’ responses to the content, comparing the information with recommendations for delivery from the US NFP National Office and from the UK FNP National Unit.

All qualitative interviews will be transcribed and analyzed thematically [57]. Interviews with women and their partners with a looked-after history will be analyzed using a computer-aided qualitative data analysis package informed by interpretive phenomenology which seeks to represent the experiences of the research participants in context [58].

### Study monitoring

Serious adverse events (any hospitalization of mother or infant other than for delivery, congenital anomaly or birth defect, persistent or significant disability, death) identified by information from participants at data collection points or using pre-paid change of circumstances cards, or from HES data at the conclusion of the study, will be recorded using the NHS National Patient Safety Agency form and reported by the chief investigator (CI) to the Multicenter Research Ethics Committee, who gave a favorable opinion within 15 days of the CI becoming aware of the event.

An independent Data Monitoring Committee (DMC) has been established, whose remit is to review the trial’s progress. Interim analyses will be supplied, in strict confidence, to the DMC, as frequently as its Chair requests. The terms of reference and a DMC charter [59] including stopping guidance will be agreed at their first meeting. Meetings of the committee will be arranged periodically, as considered appropriate by the Chair. In the light of interim data on the trial’s outcomes, adverse event data, accumulating evidence from other trials and any other relevant evidence (including updated overviews of the relevant randomized controlled trials), the DMC will inform the Trial Steering Committee (TSC) if in their view there is proof beyond reasonable doubt that the data indicate that any part of the protocol under investigation is either clearly indicated or contra-indicated, either for all or for a particular subgroup of trial participants. Unless modification or cessation of the trial is recommended by the DMC, the TSC, investigators, collaborators and administrative staff (except those who supply the confidential information) will remain blind to the results of the interim analysis. Collaborators and all others associated with the study may write to the DMC via the Trial Co-ordinating Center, to draw attention to any concern they may have about the possibility of harm arising from the treatment under study.

## Discussion

The study is dependent on local community midwifery teams identifying potentially eligible participants and asking them to sign a simple form so that their contact details can be shared with the research team. Thus, it has been necessary to develop strong links with local midwifery in each site and to tailor the collection of the contact details forms to suit the way each team works. The FNP teams also have to have close links with midwifery since they are jointly responsible for delivering midwifery care to women in the intervention arm of the trial. That has meant a substantial amount of time had been required for meetings and other liaison for the research team and for each local FNP team but it is clear that without close cooperation of all local midwives the required number of eligible women in each site with EDDs (only up to six weeks apart) will not be identified in the short time frame that is necessary. We believe that the necessary groundwork has been made and hope that the next publication about this trial will concern its results, demonstrating whether there is an impact on parenting and other aspects of maternal well-being. This is the first study to examine the effectiveness of gFNP in the UK. Results should inform decision-making about its delivery to vulnerable families not eligible for FNP, potentially enabling a wider range to benefit from the FNP curriculum approach to supporting parenting.

### Trial status

Recruitment will begin in late July 2013 and continue until January 2014.

## Abbreviations

A&E: Accident and emergency unit in hospital; CI: Chief investigator; CTU: Clinical trials unit; DCE: Discrete choice experiment; DMC: Data monitoring committee; EDD: Expected delivery date; FN: Family nurse, trained to deliver FNP according to USA NFP National office guidelines; FNP: Family nurse partnership home-visiting program (UK); GCP: Good clinical practice; GCSE: General certificate of secondary education; gFNP: Group-based family nurse partnership; HES: Hospital episode statistics; HSRU: Health services research unit; ISRCTN: International standard randomized controlled trial number; LMP: Last menstrual period; LSHTM: London school of hygiene and tropical medicine; MOS: Medical outcomes study; NICE: National institute for health and clinical excellence; NFP: Nurse family partnership, the original USA developed home visiting program, renamed FNP in the UK; NHS: National health service; NIHR: NHS national institute for health research; QALY: Quality adjusted life year; RCT: Randomized controlled trial; SOP: Standard operating procedures; TSC: Trial steering committee.

## Competing interests

None of the study investigators has any financial interests in the outcome of the trial.

## Authors’ contributions

JB conceived the study and is the chief investigator and project director. JB, EA, JBw, DE, HS, GM, EM and SP and JSs all contributed to the study design and are grant holders. EA and DE provided trials and statistical expertise and EA will supervise the randomization and conduct the primary analysis. SP and JP will conduct the economic evaluation and contributed specifically to that aspect of the paper. DA is providing practitioner input into the trial and contributed to the study design and methods. JS is the trial manager, contributing to the final selection of measures and procedures and JSs is the data manager. HS will support the integration of the trial with midwifery services. CS is co-grant holder of the embedded sub-study focusing on currently or previously ‘Looked After’ participants and will manage those qualitative methods. All authors contributed to refinement of the paper and approved the final manuscript.
